# Developing a Reablement Program Aimed at Preventing Unnecessary Care Transitions After Geriatric Rehabilitation

**DOI:** 10.1093/geroni/igab046.856

**Published:** 2021-12-17

**Authors:** Ines Mouchaers, Hilde Verbeek, Gertrudis Kempen, Jolanda van Haastregt, Ellen Vlaeyen, Geert Goderis, Silke Metzelthin

**Affiliations:** 1 Maastricht University, Maastricht, Limburg, Netherlands; 2 KU Leuven, Leuven, Vlaams-Brabant, Belgium

## Abstract

Patients returning home after geriatric rehabilitation may encounter several challenges related to daily functioning, which only manifest after returned home due to the large difference in environment and amount of support provided in both settings. This study aimed to develop an intervention preventing transitional care. A co-creation design was used, including literature research, observations, interviews, and working groups including a variety of stakeholders (n=13), including care professionals, policymakers of the municipality, client representatives, and an expert in the field of geriatric rehabilitation. Results indicated four main causes for transitional care problems: lack of communication between patients and professionals, coordination and continuity of care, patients’ limited self-management skills, and insufficient preparation. To solve these problems, an intervention was developed consisting of six intervention components aiming to increase self-management during meaningful daily activities, narrow the gap between the rehabilitation and home setting, and enhance communication and coordination.

